# Experiences with stigmatization among transgender individuals after transition: A qualitative study in the Netherlands

**DOI:** 10.1080/26895269.2020.1750529

**Published:** 2020-04-15

**Authors:** Maria J. A. Verbeek, Mark A. Hommes, Sarah E. Stutterheim, Jacques J. D. M. van Lankveld, Arjan E. R. Bos

**Affiliations:** a Faculty of Psychology, Open University, Heerlen, The Netherlands;; b Faculty of Psychology and Neuroscience, Maastricht University, Heerlen, The Netherlands

**Keywords:** gender differences, social support, status change, stigma, transgender, transition, visibility

## Abstract

***Background:*** Transgender individuals belong to one of the most stigmatized groups in society. Although the social stigma of transgender individuals has been examined many times, post transition stigma experiences among transgender individuals have received limited research attention. The aim of this study was to examine experiences with stigmatization among Dutch transgender individuals after their transition.

***Method:*** Ten trans women (age: *M* = 58.50, *SD* = 9.49) and 10 trans men (age: *M* = 42.90, *SD* = 13.62) participated in face-to-face semistructured interviews. Grounded theory was used to conceptualize and analyze the data. We examined the positive and negative reactions that transgender individuals experienced in the period after their transition. Furthermore, we explored differences between experiences of trans men and trans women. Finally, we examined differences between cisgender men and women regarding their reactions toward transgender individuals.

***Results:*** Participants reported improved psychological well-being since transition. However, they still experienced different forms of stigmatization. Trans women appeared to experience stronger social stigma than trans men. Trans women also experienced lower social status after their transition. They mainly experienced negative responses from cisgender men. Participants emphasized the importance of social and peer support.

***Conclusion:*** The current study findings demonstrate the presence of stigmatization after transition and argue for psychological aftercare. Social and peer support appeared to be important for coping with stigmatization, and improving the social network of transgender individuals is beneficial. Health providers and researchers are recommended to promote the development of constructive coping skills for transgender individuals with interventions especially targeting trans women.

## Introduction

The past decade has shown an increasing social and political concern for transgender individuals. In addition to increased media attention and political attempts to legalize the position of transgender individuals (Kuyper, 2017), there has been a positive shift in the social and cultural attitudes toward transgender individuals (Austin & Goodman, 2017).

*Transgender* is a term used for gender diverse individuals in which the common denominator is that one does not identify with traditional, dichotomous, and social constructs of gender (Budge et al., 2010; Keuzenkamp, 2012). *Cisgender* refers to a person whose gender identity matches their birth assigned gender (Green, 2006). Transgender individuals may have an ambivalent gender identity, which they express through their dissatisfaction with sex characteristics of their birth assigned sex, such as breasts, penis, and voice pitch (Kuyper, 2012; Lev, 2004). Transgender individuals can choose to start a transition process. This is a social process that can be expanded with adjustments to the body by using hormones, and with name change with the intention of being able to live in accordance with the desired gender identity (Miller & Grollman, 2015). To be able to live in their felt gender, some transgender individuals prefer a *social* transition and refrain from using hormones or other medical treatments, while modifying their appearance, clothing style and relevant entries in their passports. Others opt for a *physical* transition, and choose to undergo medical interventions, such as chest or genital surgery, or facial feminization interventions. The transition process may take months or years, depending on individual wishes and possibilities (Miller & Grollman, 2015). Dissatisfaction with one’s birth assigned sex can lead to loneliness and psychological problems (Dhejne et al., 2016; Februari, 2013; Keuzenkamp, 2012; Keuzenkamp & Kuyper, 2013; Testa et al., 2017). Furthermore, prevalence of depression, anxiety, and loneliness in transgender individuals exceeds the prevalence of these conditions in the general population (Bouman et al., 2017; Keuzenkamp, 2012; Witcomb et al., 2018), and both trans men and trans women show an increased prevalence of suicide attempts (Perez-Brumer et al., 2015).

In general, psychological problems decrease after transition (Barr et al., 2016; Dhejne et al., 2016; Keuzenkamp, 2012), as a result of increased feelings of transgender community belongingness, the strength of the awareness of one’s transgender identity, and the increased level of well-being (Barr et al., 2016). However, at the same time, the visibility of a person as a transgender individual may increase due to a discrepancy between the sex assigned at birth and the person’s gender identity. This may pose a mark on a person: a stigma is inflicted (Miller & Grollman, 2015).

Despite the positive shift in attitudes toward transgender people, many still experience stigmatization (Kuyper, 2016). Although the stigmatization of transgender individuals has been the subject of scientific research, most research attention has been paid to stigmatizing experiences of transgender individuals during their transition (Bry et al., 2018; Miller & Grollman, 2015; Norton & Herek, 2013). The stigma experiences of transgender people who consider their transition to be completed are under researched.

### Stigma

A stigma implies devaluation and rejection, and connects the marked person with socially devalued or undesirable characteristics (Goffman, 1963). Stigmatization means that someone has a known, visible, conspicuous, and discredited status (Miller & Grollman, 2015). It may evoke subtle or overt negative responses toward stigmatized individuals, and can make them vulnerable for discriminatory treatment and psychological distress (Quinn & Chaudoir, 2009; Stutterheim et al., 2011). Stigmatization is a social process that can affect lives of people with that mark (Goffman, 1963; Pescosolido & Martin, 2015), and may lead to social disapproval, status loss, and discrimination (Bos et al., 2013; Pescosolido & Martin, 2015). Several variants of stigmatization can be distinguished (Pescosolido & Martin, 2015). In this study we use the term *enacted stigma* to refer to the way individuals in society discriminate against and prejudice the stigmatized (Pescosolido & Martin, 2015). Enacted stigma includes avoidant behavior, rejection, exclusion, verbal abuse, bullying, and even physical violence (Budge, Adelson, et al., 2013; Budge, Katz-Wise, et al., 2013; Februari, 2013; Keuzenkamp, 2012; Kuyper, 2012; Miller & Grollman, 2015; Norton & Herek, 2013; Stotzer, 2009).

Another stigma variant is *anticipated stigma* which refers to individuals’ expectations of a stigmatizing experience and the belief that others assign negative attributes to them (Quinn & Chaudoir, 2009; Teh et al., 2014). Anticipated stigma, also termed “felt” stigma (Herek et al., 2009; Scambler, 2004), is the fear of enacted stigma and anxiety related to being associated with a stigmatized group. Anticipated stigma can motivate the use of self-presentation strategies to avoid stigmatization (Chaudoir & Quinn, 2016; Scambler, 2009; Teh et al., 2014). It can also lead to concealment, resulting in social identity denial and social isolation (Herek et al., 2009; Kosciw et al., 2015; Teh et al., 2014). Further, it may negatively impact psychological well-being, and can be even more disruptive than actually experiencing enacted stigma (Chaudoir & Quinn, 2016; Herek et al., 2013; Pachankis, 2007; Scambler, 2004).

Other studies distinguish *internalized stigma* or internalized transphobia in addition to anticipated stigma (Bockting, 2015; Lev, 2004; Teh et al., 2014). Internalized stigma is the individual acceptance of negative beliefs about the stigmatized identity. Internalized transphobia can be described as the discomfort with one’s transgender identity (Bockting, 2015), and may reflect impairment of one’s self-value and self-concept (Corrigan et al., 2009; Herek et al., 2009). The persistence of shame and self-hatred may also be signs of internalized transphobia (Lev, 2004). Internalized stigma or internalized transphobia can lead to lower well-being (Scandurra et al., 2018) and is associated with psychological distress, stress, and depression (Bockting et al., 2013; Breslow et al., 2015; Testa et al., 2015). Internalized stigma can also result in diminishment of one’s social network (Breslow et al., 2015), and may cause unemployment and loss of income (Keuzenkamp, 2012; Kuyper, 2017; Mizock & Mueser, 2014). Further, previous research has demonstrated that higher levels of internalized transphobia is associated with an increased likelihood of suicide attempts (Perez-Brumer et al., 2015).

Stigmatization can take place in one’s immediate surroundings, such as within the family (Riggs et al., 2015), at work (Budge et al., 2010; Schilt, 2006; Stutterheim et al., 2017; Vennix, 2010), in a health care center, in public, and in personal contact with official authorities (Keuzenkamp, 2012). Doubts about another person’s gender can induce feelings of discomfort due to a general preference for recognizable gender-normative behavior (Keuzenkamp & Kuyper, 2013; Vennix, 2013). These feelings of discomfort can give rise to stigmatizing behavior (Budge et al., 2013; Februari, 2013; Kedde & van Berlo, 2011; Keuzenkamp, 2012; Kuyper, 2012; Norton & Herek, 2013).

### Visibility

Those who are visible in public space as a transgender individual can be vulnerable to discrimination and violence (Budge et al., 2013; Corrigan et al., 2013; Miller & Grollman, 2015). Visibility, implying that a person can be classified as belonging to a stigmatized group because of his or her appearance (Berjot & Gillet, 2011; Miller & Grollman, 2015), can accentuate stigmatization as demonstrated in research among people with HIV (Stutterheim et al., 2011). Stigmatizing experiences of transgender individuals may intensify when their visibility increases (Bockting et al., 2013; Budge et al., 2013). Although visibility can increase the likelihood of stigmatization, it does not necessarily lead to psychological distress or mental health problems (Kosciw et al., 2015; Stutterheim et al., 2011). It may even have positive effects, such as increased social support (Luhtanen, 2002), and increased resilience (Kosciw et al., 2015), depending on the environment in which the transgender individual is visible. Discussing this visibility with others, such as friends or family, can also lead to higher self-esteem, lower levels of depression (Kosciw et al., 2015), and greater self-awareness, which in turn can promote mental health (Campbell & Deacon, 2006; Corrigan et al., 2013; Herek, 2007; Meyer, 2003).

### Differences between men and women

During the transition phase differences between trans men and trans women arise: most trans men are not visibly transgender due to the effects of hormone treatments, while some trans women remain visibly transgender, and may thus experience and/or perceive more stigmatization (Budge, Adelson, et al., 2013; Budge, Katz-Wise, et al., 2013; Kedde & van Berlo, 2011; Keuzenkamp, 2012; Kuyper, 2012; Stotzer, 2009). Also, research has shown a difference in the extent to which cisgender men and women stigmatize transgender individuals. For example, in a survey study conducted with lesbian, gay, bisexual, and transgender (LGBT) people in the Netherlands by Kuyper (2016), cisgender women were reported to react more positively to trans people than cisgender men.

### The present study

Earlier research on the stigmatization of transgender people has primarily focused on the first stages of transition (Austin & Goodman, 2017; Breslow et al., 2015; Keuzenkamp, 2012). The post transition period, characterized by self-acceptance of felt gender, integration in society, and living as a transgender individual (Devor, 2004), has received less attention (Kuyper, 2017). For this reason, we set out to explore, using qualitative methods, the positive and negative reactions experienced by Dutch transgender individuals after their transition. We also examined different manifestations of stigmatization and differences between the experiences of trans men and trans women. Finally, we explored differences between cisgender men and women in their reactions to transgender people.

## Method

### Study design

In this qualitative study, grounded theory (Charmaz, 2006) was used to collect and analyze the data in order to discover common and recurring themes in the data.

### Recruitment and sampling

Participants were recruited in the first phase of the study by sending e-mails to visitors of a transgender peer support group in the North of the Netherlands. Later, snowball sampling (Browne, 2005) was used to further recruit participants through participants who were included earlier in the study, and through the networks of transgender people. Participants were given information by e-mail regarding the purpose, the procedure of the interview, confidentiality, and the possibility to withdraw at any time. An informed consent statement was signed by participants immediately before the start of the interview. All participants received a pseudonym so that they could anonymously be quoted. Only participants who considered their transition to be completed participated in the interviews. The sample comprised 10 trans men (age: *M* = 42.90, *SD* = 13.62) and 10 trans women (age: *M* = 58.50, *SD* = 9.49) between the ages of 20 and 75 years. Of all the participants, 10% had completed a social transition without medical interventions, 90% had undergone both a social transition and gender affirming medical treatment, including hormonal and/or surgical interventions. Most participants identified as man or woman; some of them identified as transgender, or trans man or trans woman. At the time of the interviews, 40% of the participants had a female partner, none of the participants had a male partner. In terms of educational attainment, 30% had completed vocational education and 70% had completed college or university. A little more than half of the participants lived in an urban center (55%) and the remaining participants lived in a non-urban area (45%).

All participants considered themselves well integrated in society. Half were in some way involved in the transgender peer community by, for example, giving information to other transgender people, students, health providers, or taking part in buddy projects, being a member of a transgender peer group or organization or by representing interests of transgender individuals in other ways. Some participants preferred to be open about their transgender identity only in the *private* sphere; others openly discussed their transgender situation in *public* or presented themselves as a transgender individual in their *function* as a representative of transgender interests (Table 1).

**Table 1.** Post transition topics.

Alias	Open in	Gains due to the transition (quotes by participants)	Losses due to the transition (quotes by participants)
Johan	Public	I am fully accepted and appreciated, and I have found a place in society	Nothing
Paul	Private	I am a bit more myself and at ease	The difficulty finding a partner
Frans	Function	Life is much easier now	My old name
Joop	Public	What I see in the mirror matches the self-image that I always had	Being a woman
Fred	Public	It has made me much happier	Possibility to give birth to children
Cor	Public	I'm really free, a lot happier	My sex drive
Marti	Private	I am really satisfied with my life and before [the transition] I was not	I can't cry anymore
Lars	Private	I did not expect the impact, for the first time I was self-confident	
Rinus	Function	Self-confidence	Confided contacts with women
Sven	Public	I feel I really can be myself	My relationship
Claire	Public	I am more vulnerable and stronger, I have a lot of courage	My sex drive
Cora	Public	I won my unknown self	My dear wife
Carla	Public	I have become self-aware, and I have grown socially and emotionally	Anonymity
Jannie	Function	The transition has made me much more open	My career, making love with my wife
Solveig	Function	All the ballast is off, the transition has given a lot of space in my head	I lost myself as a guy
Babs	Function	Coming home to myself, that is the most important and essential result	
Els	Public	I feel comfortable in my body now	Nothing
Fabienne	Function	I can express myself freely the way I want to	A job
Francien	Function	I'm not that sad anymore	Being a man
Saskia	Function	I just feel better now that I can live as a woman. I am more friendly nowadays	People around me dropped out

### Ethical approval

All procedures performed in the study involving human participants were in accordance with the ethical standards of the ethical committee of the Open University of the Netherlands and with the 1964 Helsinki declaration and its later amendments or comparable ethical standards.

### Data collection

Between March 2015 and October 2015, face-to-face interviews of approximately 90 minutes were held by a female researcher with a background in LGBT research. The interviews were conducted in Dutch at a location chosen by the participant. All interviews were digitally recorded and transcribed verbatim using Dragon Naturally Speaking 12.0 speech recognition software. A semistructured protocol of questions, and follow-up probes, was designed and reviewed by a trans man working as a consultant at a mental health care center for people who struggle with their gender identity. The protocol was also pilot tested in an interview with a trans woman and then revised. The revised protocol was subsequently used during the interviews (see Appendix A). The topics covered were: life after transition, positive and negative reactions, enacted, anticipated, and internalized stigma, and the integration of transgender people. Participants were asked questions such as, “What has the transition done to you as a person?”, “What reactions did you get since your coming out?”, and “How do cisgender men and women respond to you since your transition?”

### Data processing and analysis

Data were processed using QSR NVivo 10.0 software. The transcribed interviews were coded in accordance with grounded theory (Charmaz, 2006), meaning labels were attached to segments of data describing what each segment was about. First, line-by-line coding was performed to identify emerging subcategories. Subsequently, the subcategories were combined into higher categories. The last phase of the analysis process involved deriving themes from all higher categories, namely life after transition, positive and negative reactions, social support, stigmatization, visibility, gender differences, and status change. Selected quotes were translated into English and reviewed for congruence with the original text in Dutch by the third author, who is proficient in English and Dutch.

## Results

### Life after transition

All participants reported experiencing improved psychological well-being due to their transition. They reported feeling less depressed, having fewer suicidal thoughts and more peace of mind, and described their transition as the start of a new life. The participants accepted themselves in their felt gender and were to some extent integrated in society. Most participants were open about being transgender (Table 1). Many of the participating trans women expressed happiness about wearing beautiful clothes, jewelry, and make-up, and many of the participating trans men expressed their joy related to having a flat chest, a low voice, and being able to wear swimming trunks. However, in addition to enjoying the outcomes of their transition, most participants mentioned uncertainty about living in their gender identity due to their limited knowledge about issues related to their gender.

Already in primary school, a whole lot of young girls play with each other’s hair and with their mother’s nail polish. Those of us that had our coming out later in life never had that – that learning trajectory with our sisters, with our friends, with our mother. (Cora, 66, trans woman)

Beside the “gains” after transition participants reported losses in terms of their sexuality, contact with friends, work, and their partner. In Table 1, quoted gains and losses are displayed.

I lost myself as a guy – it is so great to be with the guys in the locker room. It smells like the fresh dirt that you brush off your football shirt. A crate of beer gets brought in and then another and another. And, it’s so unbelievably fun with those guys after a game. (Solveig, 60, trans woman)

### Positive reactions

Some positive reactions, described here, referred to the beginning of the transition process. These positive reactions were considered very important for the well-being of the participants in the course of their transition and afterward, causing early and later experiences to become intertwined. A considerable number of participants received positive reactions from parents and siblings after announcing their transition. Most mothers supported and accepted the transition right away. Most fathers, however, needed more time to deal with the new situation, but, after the transition, they supported their son or daughter as well. Some close friends reacted positively to the transition and this strengthened the mutual bond, as Marti, a 20-year-old trans man, conveyed:

My relationship with my friends is better because I have become more laid back and more open, so, in general, I’m just a lot happier, and a lot more satisfied.

Positive reactions from employers during transition were also reported. These helped transgender participants in their coming out process at work and most of the participants in this study continued working in the same place after their transition. Els, a 61-year-old trans woman, said:

I first talked to the director to see how he would react and he said, ‘Does this mean you can’t do your job?’ ‘Of course I can.’ And he said, ‘What’s the problem then?’

Johan, a 66-year-old trans man, received positive reactions from his church, which resulted in a job within that church after his transition. He said:

We had a woman as our pastor then and she said something during a prayer about people walking around with secrets. So I called her up and I said, “Come and talk with me,” and she came. And I said, “Yeah, this and that.” I had it all written down and I said, “I’m going to change [transition].” And she said, “How exciting! We’re going to help you with that!” And they did. She came over to talk I don’t know how many times. After some time, I even gave a talk about it during the coffee after the service. I was so well taken care of.

Receiving social support was another positive reaction, and participants mentioned that family and friends played an important part in providing social support. Additionally, some participants reported receiving social support from fellow transgender individuals in a self-help group. Support was provided by means of exchanging experiences and tips, and undertaking activities together.

Being in a self-help group feels like coming home. It turns out there are more people like you in this world and they’ve pulled it off. Those are people where you can see that’s how it’s going be, that’s the reality. Obviously, there are some people that, like you, just signed up a few months ago and those are your “buddies” at that moment. You share experiences with each other about the operation and the hormones. All of the sudden, you start talking about, “How are you feeling?” They had never heard that from me – how I felt. That’s really unique, especially that first year when you’re doing the counselling, no hormones yet. Then you are so happy that this [the group] is possible. (Sven, 49, trans man)

Participants who identified as (trans) men also reported positive experiences regarding their social status as a man. They indicated that, after the transition, they felt more heard and seen during conversations, in contrast to their former experiences as a female assigned at birth. They experienced inclusion in the world of cisgender men. Cisgender men did not discuss emotional aspects of the transition. Cisgender women reacted to trans men with interest during and after transition, asked questions, and sought contact.

### Negative reactions and stigmatization

Unfortunately, participants also experienced stigmatization to their transgender status. Participants reported forms of enacted stigma: they, as a result of their transgender identity, were laughed and stared at, avoided and abused.

#### Enacted stigma

Enacted stigma can be in a gesture, as Jannie, a 55-year-old trans woman, described:

And then there was this woman, with her two little kids, and she saw me coming and she made a protective gesture [to keep them away from interviewee]. That really hurt.

Another participant referred to an incident in a store where she, a few years after her transition, occasionally came:

A horrible reaction on the other hand that I had in a store where I sometimes would come to buy something quite expensive. There I had the reaction that they would rather not have me come back. They had another customer that had found out that I wasn’t a woman and [that customer] said, “If that woman comes here more often, then I won’t come anymore.” (Saskia, 65, trans woman)

Some participating trans women received negative reactions from their friends, as Carla, a 50-year-old trans woman, experienced:

I come from a macho world with fast cars. We used to drive at the Nürburgring [in Germany]. I had my friends there, but not anymore. There’s no one left.

Other negative reactions came from employers who reacted with hostility at their employee’s wish to transition; they refused to support the transgender individual or looked for excuses not to continue the employment contract.

In that final meeting, it became clear that the management team didn’t think it was responsible for them to let me be in contact with the clients because they come from all sorts of cultures that don’t accept that [being a transgender person]. I asked about work performance. That was above average. The director said, “I hired you as a man, and now you’re a woman. And I don’t know what kind of quality you have as a woman.” (Claire, 40, trans woman)

The participants who experienced problems at their work during the transition, and who were fired or who resigned as a result of these problems, were often still looking for a job after transition. Some of them experienced that finding a job after transition was difficult when they revealed their transgender identity.

Once, at a temporary job, I needed to drive the bakery’s truck and deliver bread. I had my driver’s license so all was well. [They told me to] “come by, bring the paperwork, and then we’ll get everything sorted.” I came in and there were doubts. They made me leave for a bit and then [when I came back] they said, “Now, can you imagine? The baker just called and the vacancy no longer needs to be filled.” (Carla, 50, trans woman)

Some trans women indicated that cisgender men considered the transition as a descent down the hierarchical ladder. Cisgender men made more stigmatizing comments than cisgender women, seldom asked questions about the transition process and often ignored trans women. They were reported to be condescending and avoidant in conversations. Saskia, a 65-year-old trans woman reported:

Because he knew I am a trans woman, that man said, ‘I do not want to sit next to that woman.’

#### Visibility

A factor of influence in experiencing enacted stigma was visibility, suggesting that a person can be classified as belonging to the stigmatized group of transgender individuals because of his or her appearance. Trans women appeared to experience a higher frequency of enacted stigmatization experiences after their transition than trans men, who reported experiencing less enacted stigma after their transition, probably because they did not appear transgender.

Besides appearance, low voice pitch in trans women was reported as an important factor that increased the likelihood of experiencing enacted stigma. Some trans women in this study mentioned low voice pitch as contributing to experiences of post-transition *misgendering*.

There are sometimes little children that wonder, what is that? And then just ask, “Are you a man or woman because you look like a woman but you talk like a man.” “Pity! Little one, I don’t like it either but one has a high voice and the other has a low voice.” (Cora, 66, trans woman)

Both trans men and trans women considered their voice important in this respect, although the rationale behind it appeared to differ. Trans men reported the moment when they had reached a low voice as the moment when they were recognized as a man, as Lars, a 39-year-old trans man, described:

For me, it was, that I felt that I was believable [i.e., passable], that they can’t see anything feminine on me anymore and now is pretty much the moment. These past few months, my voice has also finally dropped.

In contrast, some trans women considered their voice “a revealer of their past.” Jannie, a 55-year-old trans woman, stated:

I spent two years with a speech therapist and I practiced a lot. I learned a lot there but my voice is not that great. On the phone, I’m still unfortunately often addressed as ‘sir’.

Surprisingly, some participants candidly indicated that they reacted negatively to transgender individuals of the opposite gender identity. According to some trans men, trans women were too visible “with their big hands and feet” and generated too much negative attention in the media as Johan, a 66-year-old trans man, remarked:

You do not want to be associated with them, do you?

Joop, a 53-year-old trans man said:

We were just talking about trans women. I stigmatize them. Then I say, “Wear something nice or shall I take you shopping? Act like you’re supposed to….” but then my wife always says, “They want to be precisely like you don’t want to be. Maybe that’s why you have that aversion to them”. (Joop, 53, trans man)

Some trans women gave trans men the nickname “grey mice,” because they keep a low profile.

#### Anticipated stigma

Participants also mentioned anticipated stigma after transition. Anticipated stigma, which is, in effect, the constant awareness of (potential) negative reactions was reported to be driven by fear of rejection or the belief that others engage in negative attributions. Participants indicated that they felt insecure about their appearance, leading to the fear of being outed. In fact, most participants indicated that they were always aware of reactions they might bring about in others.

It’s mostly in my mind, when I run into something. When I go swimming – my chest looks horrible – then I’m really aware of who then looks at my chest. But if someone dared to say something – then I will deal with him! (Cor, 44, trans man)

Els, a 60-year-old trans woman, stated:

That’s why I can’t be found on social media or the internet…I’ve always been careful about that because, if you apply for a job, the first thing the boss does is google. And if he sees that you’re in the board of a transgender organization, then you disappear to the bottom of the pile. There’s no way you’d get in.

Avoidance can be seen as an expression of anticipated stigma and can lead to situations such as the situation described by Lars, a 39-year-old trans man. He found it difficult that others might see aspects of his body that reflect his sex assigned at birth:

Yeah, when I go skating, I don’t get changed in the locker room; I get changed at home.

Fred, a 29-year-old trans man, spoke of his experiences during a workshop organized by his school:

At the workshop, you have to really share with each other on an emotional level. Then it’s, for me, much harder to share [that I am a trans person]. If people don’t accept it, the impact is much greater.

And also,

After my transition, I withdrew – when I was thrown out of my job. For a long time, I didn’t dare take public transit because, every time, there were negative reactions. People stared at me. There was giggling. They would turn around and look at me. (Babs, 62, trans woman)

#### Internalized stigma

Internalized stigma occurs when the stigma becomes part of one’s own value system and self-concept. Few participants reported internalizing negative perceptions about transgender individuals during the interviews. However, some participants made comments reflective of internalized stigma or spoke of internalizing negative images.

Twice, because of my hips, I was recognized as a trans man, and I really didn’t like that….and, when I was in the shower and saw myself naked, then I thought, “You know, those people are right.” It made me so sad. (Frans, 40, trans man)

Internalized stigma often comes with feelings of guilt toward others, as Sven, a 49-year-old trans man, said:

Especially with God. That’s one of the reasons why it took so long, that Calvinist sense of ‘This is the body you were given for this world so apparently that’s how it’s supposed to be. Can I go ahead and have that cut [changed]? This is after all the body God gave me.’

Devaluating oneself can also be seen as an expression of internalized stigma as Fred, a 27-year-old transman, described:

It was the whole chaos of ‘It’s not okay, it’s wrong, it’s wrong’ and, if you tell yourself that a hundred times, then it is wrong.

Internalized stigma may lead to avoidant behavior and withdrawal which may cause social isolation and loneliness as Carla, a 50-year-old trans woman, said:

I am now done with the transition, but I feel lonely.

During the interviews, the participants reflected on their transition in terms of gains and losses (Table 1), their well-being, the impact of social support, the experiences with stigmatization, visibility, the importance of the voice pitch, and the differences between men and women (Table 2).

**Table 2.** Summary of key findings.

1. The participating trans men and trans women reported improved psychological wellbeing after transition
2. They emphasized the importance of perceiving social and peer support
3. They still experienced enacted stigma after transition and reported the impact of visibility
4. They mentioned anticipated and internalized stigmatization
5. There appeared to be differences between trans men and trans women in change of social status
6. Cisgender men and cisgender women treated trans men and trans women differently

## Discussion

In this study we explored aspects of life as it is experienced by transgender people after their transition. Specifically, we explored positive and negative experiences as well as different manifestations of stigmatization experienced by Dutch transgender people. We examined differences between stigma experiences of trans men and trans women and between non-transgender men and women in their approach of transgender people. The key findings (Table 2) are consecutively discussed. The transgender people who participated in the study experienced improved psychological wellbeing and the possibility to live according to their gender identity. These findings are in line with research by Riggs et al. (2015), who, in a study among adult transgender people in Australia, found that most participants reported better mental health after transition. Most participants perceived social support from family and partners as indispensable; they reported family support as well as peer support during transition. Similarly, in their study about social support among transgender individuals, Budge et al. (2013) stated that social support appears to influence transgender individuals’ well-being and diminishes feelings of fear during and after transition. Our findings also showed that a number of participants experienced positive outcomes of peer support within the transgender community. This can be perceived as advantageous as research by Bariola et al. (2015) suggests that frequent contact with transgender peers was associated with greater resilience. Nonetheless, participants still reported experiencing enacted stigma after transition. This is in line with research conducted in the Netherlands that showed that negative reactions decrease after transition, but still occur in public spaces (Keuzenkamp, 2012). Enacted stigma was mostly reported by participating trans women, which may be due to their greater visibility as transgender, which is in line with findings from a study by Motmans et al. (2015), where they reported on experiences of violence against transgender people in Belgium. They found that visibility and stigmatization were interrelated. Some trans women reported feelings of “double” stigmatization: discrimination based on their visibility after their transition, which is intensified by discrimination based on their new gender identity as a woman, who continue to experience discrimination in society. This is supported by work by Kosumi (2017), who, in her study of discrimination of women in the private sector, stated that women continue to be discriminated against. Almost all participants mentioned being vigilant and fearing stigmatization long after the transition completed. This is reflective of anticipated stigma. Bockting et al. (2013) concluded in their study on stigma and mental health in transgender individuals, that trans women who were less open about their transgender identity also tended to have higher levels of anticipated stigma, probably because male gender nonconformity is stigmatized more often than female gender nonconformity. Experiencing anticipated stigma can be more disruptive than experiencing enacted stigma (Chaudoir & Quinn, 2016; Scambler, 2004; Scambler & Hopkins, 1986), maybe due to the fact that stigmatization itself can be ignored or denied, but *fear* of stigmatization is always carried along.

In our study, some of the participants kept their transgender identity concealed, or showed signs of guilt or withdrawal, which may be a consequence of internalized stigma. Internalization is likely to have negative effects as indicated by Pachankis (2007) in his paper on the psychological implications of stigma: concealing a stigma may lead to problems with self-monitoring, social avoidance, and difficulties starting new close relationships. Conversely, Corrigan et al. (2013) found, in their study among people with mental illness, that openness has the potential to protect against the negative effects of internalized stigma on quality of life, especially when disclosure is a step-by-step process. Such positive effects may also apply to transgender people. Transition may have serious implications for the social life of transgender people. Participating trans men reported a status change: after transition, they were taken more seriously than before by people in their environment, which is in line with findings by Schilt (2006) on the experiences of trans men in their workplace. She investigated gendered workplace inequalities from the perspective of trans men in the United States, and found that some trans men experienced more authority, prestige, and respect at work after the transition, in the absence of improved skills or ability. In contrast, trans women experienced devaluation in workplace status after transition, as was also found by Bockting et al. (2013) in their study about stigma, mental health, and resilience among transgender people in the United States. A surprising finding of our study pertained to changes in voice during and after transition. For some trans men reaching a low voice pitch was experienced as the moment they were no longer “visibly” transgender. Trans women, however, experienced their low voice pitch as the reason for misgendering. Misgendering by voice may be a problem as Pasricha et al. (2008) found in their study about communicative satisfaction among trans women. The trans women in our sample experienced the most contempt from cisgender men. This finding is in line with research findings put forth by Fisher et al. (2017), who studied the attitude of men and women toward sexual minorities and found that cisgender men showed significantly higher levels of transphobia than cisgender women. In that study, the authors described how cisgender men experienced the transition of transgender individuals as an impairment of their own masculinity (Fisher et al., 2017); they may see transgender people as a threat, and the devaluation of transgender people is a response to that threat (Norton & Herek, 2013). In this study, most participants reported openness and integration in society, which are characteristics referring to the post-transition stages of Devor (2004). The participants may have achieved these characteristics by the way they cope with stigmatization. The high educational level of most participants, may be beneficial in reflecting on one’s own behavior and may lead to a better understanding of the behavior of others. Furthermore, given that the average age of participants was over 40, it is possible that participants had developed an extensive social network during their lifetime, which may have facilitated integration, openness, and self-acceptance.

### Practical implications and recommendations

The findings of this study advance our insight in the life of transgender people after transition and may provide relevant information for transgender people, health providers, and public institutions. More specifically, our findings have shown that transgender people still experience variants of stigma after transition. Therefore, additional professional psychological support may be required in the period after transition. The introduction of programs to reduce the effects of stigmatization may therefore be beneficial. For example, an adapted version of the Coming Out Proud program, which is an intervention program for people with mental illness in the United States (Corrigan et al., 2013), might also help both trans men and trans women in reducing the effects of internalized stigma. Additionally, based on our own findings and recent publications about the impact of social connectedness among transgender people (Austin & Goodman, 2017), we recommend enhancing social and peer support for transgender individuals, as this may create resilience against stigmatization and its detrimental effects.

Further, given that almost all participants highlighted the importance of their voice pitch, we recommend paying more attention to the communicative satisfaction of transgender individuals (Pasricha et al., 2008). Link and Phelan (2001) have previously outlined that members of stigmatized groups are not merely passive recipients of stigma, but also use coping strategies to withstand stigmatization. Accordingly, in their qualitative research on emotional and coping processes throughout the gender transition, Budge, Katz-Wise, et al. (2013) found that transgender individuals face unique stressful situations, we recommend quantitative longitudinal research on the coping strategies that transgender people use to deal with these unique transgender-specific situations. Furthermore, we recommend qualitative longitudinal research on coping strategies in the successive stages of transition and on how transgender people experience social support during these stages.

This study has strengths as well as limitations. The first strength is the qualitative approach, which provides broad and in-depth understanding of the lived experiences. Qualitative research focusing on the experiences of transgender people may thus contribute to a broader understanding of the problems that transgender people face. The second strength concerns the focus on post-transition experiences, which are understudied.

This research also has limitations. First, our study was conducted using a sample of transgender people who predominantly came from the Northern part of the Netherlands. This nonrandom recruitment of participants and the limited demographic distribution may have influenced our findings. Second, half of the participants in this study were in some way active in the transgender community. In this capacity, they may have developed better coping skills to deal with stigmatization, and they may have experienced more peer support, which builds resilience against stigmatizing experiences (Bockting et al., 2013). This may explain that overall only a few participants reported internalized stigma. Another limitation of this study is the absence of participants who identify as nonbinary. The lack of participants who identify as nonbinary may have been due to the way in which the text in the recruitment mail was framed. We invited “transgender people, who believe that their transition is completed.” Non binary people may have felt that this did not apply to them.

## Conclusions

The findings of this study help to advance insight in the lives of transgender individuals after transition and provide relevant scientific knowledge for transgender individuals, health providers, and policy makers. Because participants reported stigma experiences after transition, post-transition psychological aftercare is recommended. Social support, especially peer support, appeared to be indispensable, and building and expanding a social (transgender) network may therefore be an important part of the health care professional’s task. We recommend that health care providers and researchers in collaboration with trans communities develop and test interventions to promote constructive coping skills, especially for trans women.

## Appendix A

Interview Protocol

Life after transition

What has the transition done to you as a person?

What did you gain after your transition?

What did you lose after your transition?

Positive and negative reactions

What reactions did you get since your coming out?

Are you open about being a trans man or a trans woman? If so, to what extent?

Experiences of stigma

Do you feel that transgender people are treated differently? Can you describe the feeling?

How is the social interaction with others after your transition?

Internalizing stigma

Have you felt guilty or ashamed because of the transition?

Are you aware of stigmas that might exist with regard to you?

Transgender individuals and integration

How well are transgender individuals integrated in society?

How do men and women respond to you since your transition?

How do you respond to men and women since your transition?
